# Downstaging in opportunistic breast cancer screening in Brazil: a temporal trend analysis

**DOI:** 10.1186/s12885-019-5647-8

**Published:** 2019-05-10

**Authors:** Diama Bhadra Vale, Cassio Cardoso Filho, Julia Yoriko Shinzato, Fernanda Servidoni Spreafico, Partha Basu, Luiz Carlos Zeferino

**Affiliations:** 1 Obstetrics and Gynecology Department, School of Medical Sciences of Unicamp, Rua Alexander Fleming 101, Campinas, 13083-790 Brazil; 2 Medicine School of PUC-Campinas, Av. John Boyd Dunlop s/n, Campinas, 13060-904 Brazil; 3 International Agency for Research on Cancer, 150 Cours Albert Thomas, 69372 Lyon, France

**Keywords:** Breast Neoplasms, Neoplasm Staging, Early Detection of Cancer, Epidemiologic Studies

## Abstract

**Background:**

Breast cancer is the most common female cancer in Brazil with an estimated 60 thousand new cases per year. Widespread use of mammography opportunistic screening has been observed in the last 20 years, including women under 50 years old. The present study aimed to analyse the trends in breast cancer stage distribution at diagnosis as a function of age in the study period.

**Methods:**

This paper examined temporal trends of stage distribution in women with breast cancer diagnosed between 2000 and 2015 in São Paulo state, Brazil. Data from the Hospital Cancer Registry of the region were utilized. Completeness was high. The sample was described according to age, stage and date of diagnosis using absolute frequency and proportions (%). For trends, the Cochran-Armitage test was used with a 5% level of significance (*P*-value< 0.05).

**Results:**

A total of 93,674 women were included in the analysis with a median age of 56 years old. One-third (34.4%) of the women were younger than 50 years old, and stage II was the most frequent stage (36.4%), even when analysed by age groups. Stage 0 corresponded to 7.7% (7247 women) of cases. In the study period, there was a significant trend towards an increase in Stages 0, I and IV (*P* < 0.01) and a trend towards a decrease in Stages IIA, IIB and IIIB (*P* < 0.001). Stage IIA was more prevalent until 2009, and stage I was more prevalent thereafter. The trends to increase the proportion of Stages 0 and I and to decrease the proportion of stages IIA, IIB and IIIB were significant in all age groups.

**Conclusions:**

Breast cancer cases are diagnosed mainly at early stages, and approximately one-third of cases are younger than 50 years old. Downstaging has been shown. Opportunistic screening may have supported these results. Further studies are needed to show whether these results will impact the prognosis.

## Background

Measuring changes in cancer burden is an important parameter to evaluate cancer control programmes. Breast cancer is the most common cancer and the leading cause of cancer death among women worldwide [1]. Screening has contributed significantly to reduce cancer mortality, especially in countries that implemented population-based well-organized screening programmes [2]. An intermediate indicator of breast cancer screening effectiveness is the downstaging of the disease in the screened population. Tracking the stage at diagnosis over time can help to understand the impact of screening.

Early detection of breast cancer aims to identify the cancer at an early, often curable stage. Differences in breast cancer survival are observed between stage I and II but are more evident between stages II and III or IV [3, 4]. Mammography screening is capable of detecting the cancer at a size between 0.2 and 1.0 cm, when the cure rate is as high as that in microinvasive carcinoma (≤ 0.1 cm) [5]. There is sufficient evidence that breast cancer screening in women from 50 to 69 years old reduces mortality rates [2]. The evidence favouring screening of women under 50 years old is considered to be limited [2].

In Brazil, a national guideline recommends to screen women between 50 and 69 years old every two years with mammography [6]. In the absence of a system to identify eligible women individually and invite them to screening, the programme is opportunistic. In São Paulo state, widespread use of mammography screening has been observed in the last 20 years due to incentive policies. In 2000, approximately 441,000 mammograms were performed in the public health system, and the number increased by three-fold to 1,307,000 mammograms in 2012 [7]. From 2000 to 2015, there was an increase in the female population of the state by 29% in the 40–49 age group, 74% in the 50–59 age group and 81% in the 60–69 age group [8]. Screening under 50 years of age is very common and represents approximately 20% of all mammograms performed [9]. The estimated incidence rate per 100,000 women in Sao Paulo state was 42 in 2002 and 58 in 2018 regardless of age [10, 11]. Diagnosis and treatment are usually performed at the cancer centres. Health care in Brazil is free of charge, although approximately 40% of São Paulo population co-uses private care [12].

The present study aimed to analyse the trends in breast cancer stage distribution at diagnosis as a function of age in São Paulo state during the period when opportunistic screening was highly prevalent. The results can help policy makers monitoring the implementation of cancer control interventions.

## Methods

This was a hospital-based study of temporal trends of stage distribution of the women with breast cancer diagnosed between 2000 and 2015 in São Paulo state, Brazil. The data were accessed online in July 2017 from the Hospital-based cancer registry system (HBCR) managed by the São Paulo Cancer Center Foundation (FOSP) [13].

In the HBCR, the women were anonymized, and a number of variables were available in every case. We selected female cases coded as C-50 (breast cancer) according to the International Classification of Diseases, 10th edition (ICD-10). The variables age, stage and date of diagnosis were selected for analysis. The data in HBCR were provided by trained hospital technicians who regularly review cases from records and enter data into an online platform. All hospitals licensed for cancer care in São Paulo state are required to provide data to the HBCR. To avoid the duplication of cases, we included only analytical cases, i.e., cases from a particular hospital registered for primary treatment. Only cases for which the stage at diagnosis was indicated were included in the main analysis. Completeness was high. Non-staged cases corresponded to 2.3% of total cases.

Cases were staged and recorded according to the American Joint Committee on Cancer based on the TNM Classification of Malignant Tumours system (TNM). Stage IIIC started to be registered in the HBCR after 2006. To standardize the data, we aggregated cases from stage IIIB and IIIC as stage IIIB.

Variables were described by absolute frequency and proportions (%). For trends, the Cochran-Armitage test was used with a 5% level of significance (*P*-value< 0.05). A positive (+) statistic test (Z) meant a trend to increase, and a negative (−) test indicated a trend to decrease. SAS System for Windows (Statistical Analysis System), version 9.2 was used to perform the analysis.

The Ethics Committee of the State University of Campinas under the number CAAE 89399018.2.0000.5404 approved this study. The Committee waived the need for consent.

## Results

The total sample included 93,674 women with a median age of 56.15 (SD 13,51) years old. The distribution of the cases by age and stage is reported in Table 1. Most women were between 50 to 59 years old. Approximately one-third (32,254 women or 34.4%) were younger than 50 years old. Stage IIA was the most prevalent Stage in all age groups. Stage 0 was less prevalent in women under 40 or older than 69 years old.

**Table 1:** Distribution of breast cancer cases in women as a function of age and stage at diagnosis from 2000 to 2015 in São Paulo/Brazil

Group Age /Stage	< 40 years	40–49 years	50–59 years	60–69 years	≥ 70 years	Total
	n (%)	n (%)	n (%)	n (%)	n (%)	n (%)
Stage 0	477	2095	2183	1559	933	7247
	(5.0)	(9.2)	(8.7)	(8.0)	(5.6)	(7.7)
Stage I	1297	4384	5642	4909	3715	19,947
	(13.6)	(19.3)	(22.5)	(25.0)	(22.2)	(21.3)
Stage IIA	2008	5065	5638	4651	3960	21,322
	(21.0)	(22.3)	(22.5)	(23.7)	(23.7)	(22.8)
Stage IIB	1594	3336	3346	2336	2110	12,722
	(16.7)	(14.7)	(13.3)	(11.9)	(12.6)	(13.6)
Stage IIIA	1757	3241	2998	1935	1332	11,263
	(18.4)	(14.3)	(12.0)	(9.9)	(8.0)	(12.0)
Stage IIIB	1419	2878	3227	2530	2914	12,968
	(14.9)	(12.7)	(12.9)	(12.9)	(17.4)	(13.8)
Stage IV	994	1709	2057	1687	1758	8205
	(10.4)	(7.5)	(8.2)	(8.6)	(10.5)	(8.8)
Total	9546	22,708	25,091	19,607	16,722	93,674
	(100.0)	(100.0)	(100.0)	(100.0)	(100.0)	(100.0)

**Fig. 1:**
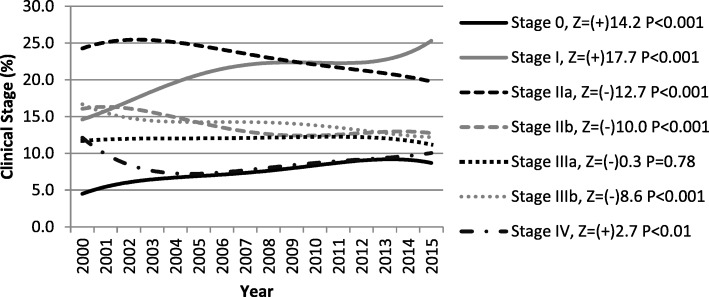
Time trends of the proportion of breast cancer stage from 2000 to 2015, São Paulo/Brazil. Legend: Z – Cochran–Armitage test for trend; (+) trend to increase; (−) trend to decrease. P = *P*-value

During the study period, there was significant trend towards an increase in Stages 0, I and IV (*P* < 0.01) and a trend towards a decrease in Stages IIA, IIB and IIIB (*P* < 0.001) (Fig. 1). Stage IIA was more prevalent until 2009, and Stage I was more prevalent thereafter.

**Fig. 2:**
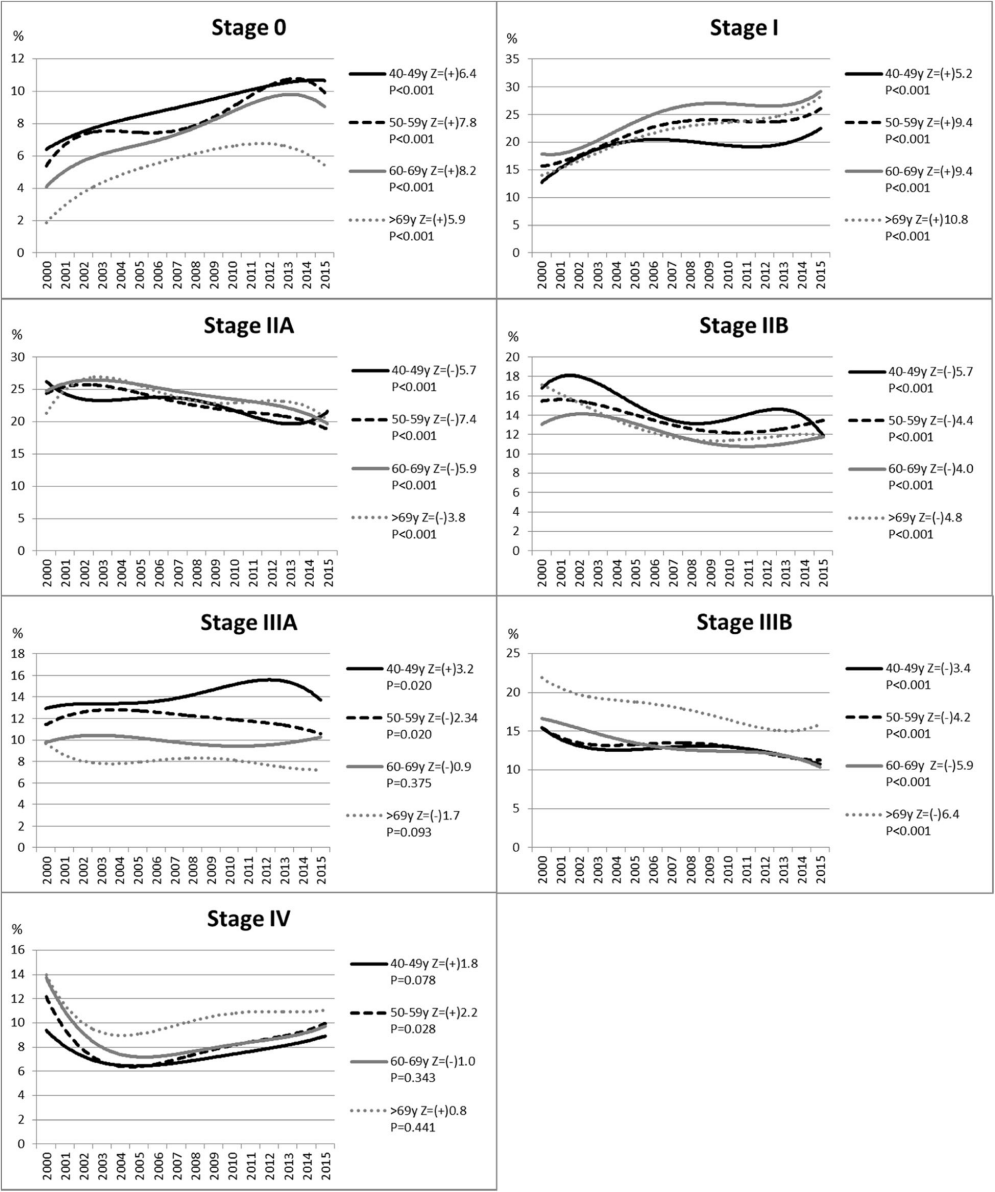
Time trends of the proportion of breast cancer stage by age-groups, from 2000 to 2015, São Paulo/Brazil. Legend: Z – Cochran–Armitage test for trend; (+) trend to increase; (−) trend to decrease. P = P-value

Figure 2 shows the trends of the proportion of cases by stage as a function of age group. The trend to increase the proportion of Stages 0 and I was statistically significant in all age groups. The trend to decrease the proportion of stages IIA, IIB and IIIB was also significant in all age groups. There was a statistically significant trend to increase stage IV in the 50–59 years old age group.

## Discussion

From 2000 to 2015, the majority of the breast cancer cases were diagnosed at localized stages in São Paulo, Brazil. We observed a reduction in the proportion of cases diagnosed at a late stage in the period. There was also a trend showing an increase in stage 0 in all age groups. These results occur simultaneously with the widespread use of opportunistic mammography screening in the region [7]. However, the impact of the large volume of screening and the change in the stage distribution on the reduction of breast cancer mortality over time needs to be studied further [14, 15].

The World Health Organization (WHO) has recommended mammography as the exclusive screening modality in the countries with adequate resources to implement such a programme with appropriate quality assurance. Early diagnosis of symptomatic women is a strategy, particularly in low- and middle-income countries where the disease is diagnosed in late stages and resources are very limited [16]. An increase in the proportion of breast cancers detected at an early stage is commonly referred to as downstaging [17]. In our study, 64,7% of the women were diagnosed at early stages (stages I or II). This finding is similar to that observed in high-income countries and other countries from Latin America [18–21]. However, the stage shift may be the consequence of large-scale screening mammography and/or better access to care.

The significant increase in the proportion of carcinoma in situ (CIS) consistently observed across the age groups is most likely due to mammography screening. It is also important to monitor the proportion of CIS among the total breast cancers detected. The European Breast Cancer Screening Guidelines recommend that the proportion should not exceed 15% [22]. In Brazil, the proportion of CIS remains below this level. Mammography screening has the inherent risk of *‘overdiagnosis’* due to the detection of early breast cancers that would not have caused any symptoms during the lifetime of the women through screening. The Independent United Kingdom Panel on Breast Cancer Screening estimates that for every 10,000 women in the United Kingdom aged 50 years attending screening for the next 20 years, 129 breast cancers would be over-diagnosed; however, screening would prevent 43 deaths from breast cancer [23].

In Brazil, screening is opportunistic due to the lack of an invitation system and a weak organization infrastructure. Most of the evidence about the effectiveness of mammography screening on the reduction of breast cancer mortality comes from population-based organized programmes [24–27]. It is important to convert the current opportunistic programme in Brazil to a population-based organized programme with an effective call-recall system, implement a screening registry and ensure robust quality assurance at all levels. In fact, the European experience has demonstrated that it is much more challenging to convert an opportunistic programme to a population-based programme compared with initiating a new population-based programme [28]. An organized mammography programme will be much more effective, minimize the risk of harm and ensure utilization of resources in a more cost-effective manner.

Approximately one-third (34.4%) of cases in this study occurred in women younger than 50 years old, which is similar to that was observed in 2000–2013 in the population-based cancer registry of São Paulo city, the capital of the state (32,9%) [29]. In the United States of America, only 20.0% of cases were in this age-group with a median age of approximately 62 years old [30]. The median age is 56.2 years in this study and 55.5 years in Mexico [31].

This difference can be due to the higher proportion of women in the younger age group, a different genetic profile observed in Latin women or an effect of the widespread use of mammography opportunistic screening in women under 50 years in Brazil. Of note, 28.5% of cancers in women from 40 to 49 years old were diagnosed in stages 0 and I.

Quality of data in low and middle-income countries is low, so it is difficult to establish evidence to better planning cancer control actions. This study was facilitated by the high completeness of data in the hospital based cancer registry of São Paulo, a high-middle income state in Brazil. The registry of more than 90,000 breast cancer cases allowed analysis with statistical significance to be performed. The main limitation is regarding the non-population based nature of the registry, preventing estimation of incidence rates. Population-based cancer registries are not commonly available in the region for analysis of temporal prevention trends. Furthermore, we do not know whether cancers in the registry were detected by screening. The relation of these findings with mammographic screening cited in the discussion is a hypothesis given that screening data are weak. Efforts should be made to improve monitoring of screening.

## Conclusions

During the study period, a clear downstaging of breast cancers could be observed probably due to a substantial increase in the use of (opportunistic) mammography screening. Breast cancer cases were diagnosed mainly at early stages, and approximately one-third of cases were diagnosed in women under 50 years old. We expect that these results will support further actions from the Ministry of Health to improve the quality and organization of the programme.

## Abbreviations

CIS: Carcinoma in situ
FOSP: São Paulo Cancer Center Foundation
HBCR: Hospital-based cancer registry system
ICD-10: International Classification of Diseases, 10th edition
TNM: TNM Classification of Malignant Tumours system
WHO: World Health Organization
